# Characterization and Regulation of the Osmolyte Betaine Synthesizing Enzymes GSMT and SDMT from Halophilic Methanogen *Methanohalophilus portucalensis*

**DOI:** 10.1371/journal.pone.0025090

**Published:** 2011-09-20

**Authors:** Shu-Jung Lai, Mei-Chin Lai

**Affiliations:** Department of Life Sciences, National Chung Hsing University, Taichung, Taiwan, Republic of China; University of Groningen, The Netherlands

## Abstract

The halophilic methanoarchaeon *Methanohalophilus portucalensis* can synthesize the osmolyte betaine *de novo* in response to extracellular salt stress. Betaine is generated by the stepwise methylation of glycine to form sarcosine, N, N-dimethylglycine and betaine by using S-adenosyl-L-methionine (AdoMet) as the methyl donor. The complete gene cluster of *Mpgsmt-sdmt* was cloned from Southern hybridization and heterologous expressed in *E. coli* respectively. The recombinant MpGSMT and MpSDMT both retained their *in vivo* functional activities in *E. coli* BL21(DE3)RIL to synthesize and accumulate betaine and conferred elevated survival ability in betaine transport deficient mutant *E. coli* MKH13 under high salt stress. The dramatic activating effects of sodium and potassium ions on the *in vitro* methyltransferase activities of MpGSMT, but not MpSDMT or bacterial GSMT and SDMT, revealed that GSMT from halophilic methanoarchaeon possesses novel regulate mechanism in betaine biosynthesis pathway. The circular dichroism spectra showed the fluctuated peaks at 206 nm were detected in the MpGSMT under various concentrations of potassium or sodium ions. This fluctuated difference may cause by a change in the β-turn structure located at the conserved glycine- and sarcosine-binding residue Arg167 of MpGSMT. The analytical ultracentrifugation analysis indicated that the monomer MpGSMT switched to dimeric form increased from 7.6% to 70% with KCl concentration increased from 0 to 2.0 M. The level of potassium and sodium ions may modulate the substrate binding activity of MpGSMT through the conformational change. Additionally, MpGSMT showed a strong end product, betaine, inhibitory effect and was more sensitive to the inhibitor AdoHcy. The above results indicated that the first enzymatic step involved in synthesizing the osmolyte betaine in halophilic archaea, namely, GSMT, may also play a major role in coupling the salt-in and compatible solute (osmolyte) osmoadaptative strategies in halophilic methanogens for adapting to high salt environments.

## Introduction

Betaine is a common compatible solute (osmolyte) that plays a crucial function as an osmoprotectant in plants, animals, bacteria and archaea [1]–[3]. Most heterotrophic bacteria transport choline into cells and form betaine using two oxidation steps: choline dehydrogenase and betaine aldehyde dehydrogenase [4]. Many methanogens can also transport and accumulate betaine in response to osmotic stress [5]–[9]. *Methanohalophilus* strains have been isolated from hypersaline environments and can grow over a range of NaCl concentrations from 1.7 to 4.4 M [10], [11]. These extremely halophilic methanogens synthesize three zwitterionic compounds, β-glutamine, N^ε^-acetyl-β-lysine, and betaine (0.6–4.1 M), and also accumulate potassium ions (0.2–3.0 M) as an inorganic osmolyte to balance external and internal osmotic pressures [12], [13]. In the halophilic methanoarchaeon *Methanohalophilus portucalensis* strain FDF1^T^, NMR analyses and both *in vivo* and *in vitro* radiometric betaine formation assays demonstrated the betaine biosynthetic pathway functions through the stepwise methylation of glycine with AdoMet as the methyl donor. In this pathway, sarcosine and N, N-dimethylglycine serve as intermediates for betaine synthesis [5], [14]. Some halophilic bacteria, for example, *Actinopolyspora halophila*, *Halorhodospira halochloris*, *Aphanothece halophytica*, *Synechococcus* sp. WH8102, and *Myxococcus xanthus*, were later shown to also demonstrate *de novo* betaine synthesis from glycine [15]–[20].

A novel broad substrate methyltransferase, glycine sarcosine dimethylglycine methyltransferase (GSDMT), purified from the halophilic methanoarchaeon *M. portucalensis* strain FDF1^T^ has been shown to possess glycine N-methyltransferase (GMT), sarcosine N-methyltransferase (SMT) and dimethylglycine N-methyltransferase (DMT) activities [5], [21]. The methyl transfer activities of GSDMT require KCl, and the internal concentration of K^+^ regulates GSDMT activities [5], [21]. In contrast, methyltransferases from halophilic bacteria have narrower substrate spectra that synthesize betaine from glycine by two methyltransferases, glycine sarcosine methyltransferase (GSMT) and sarcosine dimethylglycine methyltransferase (SDMT) or dimethylglycine methyltransferase (DMT) [15], [16], [18], [20], and both bacterial GSMT and SDMT are inhibited by NaCl and KCl [20].

Recently, another N-methyltransferase, sarcosine dimethylglycine methyltransferase (SDMT), which is also responsible for betaine *de novo* synthesis in *M. portucalensis* strain FDF1^T^, was purified and characterized [22]. This finding indicated that halophilic methanoarchaea have more than one set of betaine *de novo* synthesis systems to ensure survival in hypersaline environments. Both the SMT and DMT activities of SDMT from halophilic methanoarchaea are not inhibited by KCl or NaCl (0.2–1.0 M). Betaine (2.0 M) did not affect the SMT activity, but slightly repressed the DMT activity (65.0% residual activity left) [22]. The catalytic efficiency of SDMT is higher than the broad substrate spectrum GSDMT, which indicates that the GSMT/SDMT system may be responsible for more immediate osmotic stresses. In this report, *M. portucalensis* genes for the glycine sarcosine methyltransferase (*gsmt*) and the sarcosine dimethylglycine methyltransferase (*sdmt*) were isolated and sequenced. Due to lack of genetic and expression systems in halophilic methanogen, the GSMT and SDMT were heterologously expressed in *E. coli* for further study. The enzyme kinetics of the over-expressed MpGSMT and MpSDMT were investigated and showed that the methyltransferase activities of MpGSMT, but not MpSDMT, were modulated by potassium, sodium and betaine levels.

## Materials and Methods

### Organisms, vectors and growth conditions

The archaeal strain used in this study is *Methanohalophilus portucalensis* strain FDF1^T^ ( = DSM 7471) [10]. Cells were routinely cultured in defined medium containing 120 g l^−1^ NaCl and 20 mM trimethylamine. Trimethylamine was the sole carbon and energy source [12]. Sterile medium was prepared under a N_2_∶CO_2_ atmosphere (4∶1) by a modification of the Hungate technique [23]. Medium was anaerobically dispensed into serum bottles that were then sealed with butyl rubber stoppers and aluminum crimps (Belleco, Inc., Vineland, NJ). The methanogenic substrate trimethylamine and reducing agent Na_2_S⋅9H_2_O were added to the sterile medium just prior to cell inoculations. Sealed serum bottles were inoculated with a 0.5% volume of late exponential phase culture using an N_2_-flushed syringe. Cells were grown at 37°C as previously described [24]. Cell growth rates were monitored by removing 1 ml of the culture with a N_2_-flushed syringe into a Na_2_S_2_O_3_-containing cuvette. Optical densities were measured at 540 nm.

The pGEM®-T vector (Promega, Madison, WI, USA) was used to clone the *Mpgsmt-sdmt* gene fragment, which was PCR amplified from *M. portucalensis* chromosomal DNA. The pUC18 cloning vector (Fermentas International Inc., Canada) was used for cloning the *Mpgsmt-sdmt* and its upstream gene fragment. Both were separately transformed into *E. coli* JM101, which was cultured in Luria-Bertani (LB) medium or on solid medium containing ampicillin (100 µg ml^−1^) at 37°C. The expression vector pET28a was used to clone the *Mpgsmt* and *Mpsdmt* genes. *E. coli* BL21(DE3)RIL were transformed by these modified vectors and were grown in LB medium containing kanamycin (50 µg ml^−1^) and chloramphenicol (34 µg ml^−1^). For protein overexpression, MpGSMT and MpSDMT were induced with IPTG (1 mM). The cloning vector pUHE21, which can heterologously express its target gene in *E. coli* MKH13, was used to clone the *Mpgsmt-sdmt* gene cluster into a *Bam*HI-*Xba*I cloning site. This construct was transformed into *E. coli* MKH13 (Δ*putP*Δ*betTIBA*Δ*proP*Δ*proU*) and cultured in M9 minimal medium with ampicillin (100 µg ml^−1^) and spectinomycin (50 µg ml^−1^) for 16 h (37°C). For salt tolerance tests, *E. coli* MKH13 cells with or without the *Mpgsmt-sdmt* genes were grown in varying NaCl concentrations (0–0.8 M) at 37°C for 20 to 170 h.

### Cloning *Mpgsmt* and *Mpsdmt*

The *Mpgsmt-sdmt* complete gene cluster was obtained through Southern hybridization using a 231 bp partial *Mpsdmt* gene fragment as a probe. For PCR, the template was *M. portucalensis* chromosomal DNA, and the primers were Mpsdmt-f, 5′-TCCGAAAACCAGAAAACCGC-3′ and 2MI-r, 5′-CCWCCRTAWCCKGCWCCMAKATCMA-3′ (W, A or T; R, G or A; K, G or T; M, A or C). Labeling was performed using the DIG DNA Labeling and Detection Kit (Roche Diagnostics GmbH, D-68298, Mannheim, Germany). The forward primer Mpsdmt-f was designed from N-terminal amino acid sequences of MpSDMT [22], and the reverse primer 2MI-r was designed from the conserved amino acid sequences in the AdoMet-binding motif I of *H. halochloris* SDMT (AAF87203), *A. halophytica* DMT (AB094498), and *A. halophila* GSMT-SDMT (AAF87204). The 2.7 kb gene fragment recovered and purified from the band with a positive signal after Southern hybridization was ligated into pUC18 and transformed into *E. coli* JM101. This 2.7 kb insert was further sequenced using an ABI-PRISM 310 Genetic Analyzer. Sequencing results were analyzed using the BLASTX program from NCBI. The amino acid sequence was translated using the SDSC workbench (workbench.sdsc.edu/) and analyzed by SeqMan (DNASTAR). The partial S-adenosyl homocysteine hydrolyase (*sahh*) gene was detected in this 2.7 kb gene fragment, and a Southern hybridization was further performed using *Mpsahh∼sdmt-p2.7* as a probe. The obtained 3.9 kb gene fragment contained the glycine sarcosine methyltransferase (*gsmt*), sarcosine dimethylglycine methyltransferase (*sdmt*), S-adenosyl homocysteine hydrolyase (*sahh*) and the upstream partial S-adenosylmethionine synthetase (*sams*) genes. These were purified, ligated into pUC18, transformed into *E. coli* JM101, further sequenced by the ABI-PRISM 310 Genetic Analyzer and analyzed by BLASTX.

### Transcript analysis by the reverse transcript polymerase chain reaction

The total RNA of *M. portucalensis* was prepared as described before [24]. Briefly, mid-log phase cells (60 ml, A_540_ 0.6∼0.8, about 10^9^ cells) were harvested, lysed in 1 ml of TRI reagent (Applied Biosystems, Life Technologies, Foster City, CA) and incubated for 5 min at room temperature. After the addition of 0.3 ml of chloroform, the mixture was shaken vigorously for 15 s, further incubated on ice for 5 min and centrifuged at 12,000 *g* for 10 min (4°C). The aqueous phase, which contained the RNA, was transferred into a new tube, and the nucleic acids were precipitated with isopropanol. The RNA pellet was dried by Speed Vac (Savant Instrument, Inc., New York, USA) and then dissolved in 20 µl DEPC-treated water. Total RNA concentrations were determined by absorbances at 260 nm. DNA contamination was removed from the RNA preparation by DNase I (QIAGEN, Valencia, CA) digestion at 37°C for 1 h. The RNA was then incubated at 65°C to inactivate the DNase I. The TaKaRa RNA PCR kit (TAKARA BIO Inc., Shiga, Japan) was used for RT-PCR reactions with specific primers: Mpgsmt/NdeI-f, 5′-CATAT GATGA ACCAA TACGG AAAA-3′ and Mpgsmt-rw, 5′-AATGG GCATG GGAAT A-3′ for amplifying the partial *Mpgsmt* (530 bp); and primers Mpsdmt/NdeI-f, 5′-CATAT GATGT CTGAA AACCA AAAAA C-3′ and Mpsdmt/XhoI-r, 5′-CTCGA GTTTT TTACG TAAAT GGAA-3′ for *Mpsdmt* (837 bp), and primers Mpgsmt/NdeI-f and Mpsdmt/XhoI-r for the *Mpgsmt-sdmt* (1653 bp) gene fragments.

### Heterologous expression and purification of MpGSMT and MpSDMT

*Mpgsmt* and *Mpsdmt* were amplified separately from the pUC18-*Mpsahh∼sdmt*-p2.7 plasmid that contained *Mpgsmt-sdmt*. The complete *Mpgsmt* sequence was amplified with the Mpgsmt/NdeI-f and Mpgsmt/*Bam*HI-r primer set. *Mpsdmt* was amplified with Mpsdmt/NdeI-f and Mpsdmt/XhoI-r. Both PCR products were subcloned into pET28a at the *Nde*I-*Bam*HI or *Nde*I-*Xho*I restriction sites respectively, transformed into *E. coli* BL21(DE3)RIL, further cultured in LB medium containing kanamycin (50 µg ml^−1^) and chloramphenicol (34 µg ml^−1^) and induced with 1 mM IPTG. The *Mpgsmt-sdmt* gene cluster was PCR amplified with the specific primers Mpgsmt/NdeI-f and Mpsdmt/XhoI-r, ligated into the pET28a vector and transformed into *E. coli* BL21(DE3)RIL for *in vivo* analyses of betaine production.

Recombinant *E. coli* strains containing *Mpgsmt* or *Mpsdmt* in the pET28a expression vector were cultured in LB medium containing kanamycin (50 µg ml^−1^) and chloramphenicol (34 µg ml^−1^) until the A_600_ reached 0.6–0.8 and then induced with 1 mM IPTG overnight at 30°C and for 4 h at 37°C, respectively. Cells were harvested by centrifugation at 10,410 *g* for 10 min and suspended in buffer A (50 mM Tris (pH 7.3), 1 mM EDTA, and 1 mM 2-mercaptoethanol). Lysozyme (1 mg ml^−1^) was then added, and the mixture was incubated on ice for 30 min. Total cell extracts were obtained by sonication (total of 15 min, 6 s bursts at 300 W with 10 s cooling periods between each burst) on ice using an Ultrasonic VXC750 sonicator. Cell debris were discarded after centrifugation at 10,000 *g* for 10 min. Protein concentrations of supernatants and cell extracts were determined by a modified Bradford protein assay [25] using bovine serum albumin (Sigma-Aldrich, MO, USA) as a standard.

All purification steps were performed at 4°C. Crude extracts were loaded onto a Poly-Prep chromatography column (0.8×4 cm, Bio-Rad Laboratories, Hercules, CA) containing 2 ml of 50% Ni Sepharose™ 6 Fast Flow resin (Amersham, GE Healthcare, Quebec, Canada). Proteins were eluted using step gradients of buffer A (50 mM Tris (pH 7.3), 1 mM EDTA, 1 mM BME) containing 50 mM, 150 mM and 250 mM of imidazole in each step. The partial purified MpGSMT and MpSDMT were separated by 12.5% SDS-PAGE, and a PageRuler™ pre-stained protein ladder (Fermentas Canada Inc., Ontario, Canada) was used for molecular weight markers. Gels were stained with Coomassie blue.

### Methyltransferase activity assay

GSMT possesses glycine N-methyltransferase (GMT) and sarcosine N-methyltransferase (SMT) activities, and SDMT possesses sarcosine N-methyltransferase (SMT) and dimethylglycine N-methyltransferase (DMT) activities. GMT, SMT and DMT activities were determined in the forward direction for sarcosine, N, N-dimethylglycine and betaine formation using glycine (Sigma), sarcosine (Sigma) and N,N-dimethylglycine (Sigma) as respective substrates. An acid-washed charcoal method [5], [21], [22] was used in this study to detect methyltransferase activities. The reaction mixture contained 100 mM TES (pH 7.3), 1.0 M glycine, sarcosine or N, N-dimethylglycine as substrate and 1.0 mM AdoMet (containing [methyl ^3^H]-AdoMet 0.1 µCi 2.0 pmol^−1^, 1 Ci = 3.7×10^10^ Bq, PerkinElmer, CT, USA) as the methyl donor. Various concentrations of protein, glycine, sarcosine, dimethylglycine, betaine, NaCl and KCl concentrations were added to the reaction mixture as indicated (final volume = 100 µl). Incubations were carried out for 1 h (37°C). Reactions were stopped by the addition of 25 µl of cold 10% trichloroacetic acid followed by 125 µl of a charcoal suspension (76 mg charcoal per 1 ml in 0.1 N acetic acid). After 15 min of incubation at 0°C and centrifugation at 17,968 *g* for 10 min, unreacted [methyl ^3^H]-AdoMet was absorbed by acid-washed charcoal and precipitated. A hundred microliters of the supernatant was added to 3 ml of counting scintillant (PerkinElmer). Radioactivity was measured using a liquid scintillation spectrometer (Pharmacia Co.). All assays were carried out in triplicate. Blank values obtained in the absence of substrate (glycine, sarcosine or N,N-dimethylglycine) and in the absence of protein were subtracted to give net counts for methylated product synthesis. Enzymatic activities were expressed as nmol of transferred ^3^H-methyl group per µg of enzyme in 1 min.

### *In vivo* betaine formation

*E. coli* BL21(DE3)RIL-pET28a-*Mpgsmt-sdmt* intracellular solutes were prepared by 70% ethanol extraction. Cells were cultured in LB medium containing kanamycin (50 µg ml^−1^), chloramphenicol (34 µg ml^−1^) and 1 mM IPTG. Cells were harvested by centrifugation at 10,410 *g* for 10 min (4°C). Pellets were resuspended in 70% ethanol and incubated at 65°C for 5 min. Cell debris were harvested by centrifugation at 10,410 *g* for 10 min at room temperature. Ethanol extracts were collected twice from each sample. Ethanol extracts from *E. coli* BL21(DE3)RIL-pET28a were used as a negative control. All ethanol extracts were dried by Speed Vac (Savant) and then dissolved in 20 µl of 70% ethanol. The ethanol extracts were spotted onto TLC plates (silica gel 60, Merck, NJ, USA) with glycine, sarcosine, dimethylglycine, and betaine used as standards. Samples were separated with a phenol-water (4∶1) solvent system and colored with a 0.1% bromocresol green solution [5].

### Northern hybridization

To confirm cotranscription of *Mpgsmt* and *Mpsdmt*, *M. portucalensis* FDF1^T^ was cultured in defined medium using an optimal salinity (2.1 M NaCl) and 20 mM trimethylamine as catabolic substrate at temperature (37°C). Early log phase cultures (OD_540_ about 0.35∼0.40) were harvested in centrifugation tubes with 100 ml of culture per tube in an anaerobic Coy chamber. During the temperature stress test, the 100 ml cultures were incubated at 20 and 29°C as the cold stress and at 41 and 45°C as the heat stress conditions for 1 h. To examine whether the *Mpgsmt-sdmt* transcription level was affected by betaine, a final concentration of 0.5 mM betaine was added at the same time under the temperature stress for 1 h. The cells were harvested by centrifugation (12,100 *g*, 4°C, 15 min), and the total RNA was extracted by TRI reagent. *M. portucalensis* FDF1^T^ total RNAs (30 µg each) from different temperature stresses were loaded onto a 1% agarose gel that contained formaldehyde as the denaturing reagent. After electrophoresis (60 V, 130 mA, 2.5 h), the RNA samples were transferred onto a Hybond-N^+^ membrane (Amersham) by capillarity and hybridized with a probe for *Mpgsmt*, *Mpsdmt* or *Mpgsmt-sdmt* at 45°C for 12∼16 h. Signals were measured using TINA software and were compared to 16S rRNA signals from 5 µg of total RNA on a 1% agarose gel stained by ethidium bromide.

### Secondary structure stability experiments by Circular Dichroism Spectrometry

Circular dichrosim (CD) spectrometry was performed using a Jasco J-815 spectropolarimeter as described by Su et al [26]. CD spectra of the MpGSMT protein were recorded at 25°C, using 0.1-cm quartz cuvettes with a wavelength range of 190–250 nm at a step size of 0.2 nm. Signal averaging time was 0.25 s and the slit bandwidth was 1 nm. All spectra were corrected for the buffer absorption. The mean residue ellipticity ([θ]) at each wavelength was calculated from Eq.
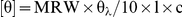
where MRW is the mean residue weight, θ_λ_ is the measured ellipticity in degree at wavelength λ, l is the cuvette pathlength (0.1 cm), and c is the protein concentration in g/mL.

### Quaternary structure analysis of MpGSMT by analytical ultracentrifugation

Sedimentation velocity experiments of MpGSMT were carried out using a Beckman Optima XL-A analytical ultracentrifuge as described by Su et al [26]. Samples (380 µl) and buffer solutions (400 µl) were loaded into the double sector centerpiece separately and built up in a Beckman An-50 Ti rotor. Experiments were performed at 20°C and a rotor speed of 42,000 rpm with 0.70 mg/ml MpGSMT in 0.1 M TES buffer (pH 7.3) contained 0 to 2.0 M KCl. Protein samples were monitored by UV absorbance at 280 nm in a continuous mode with a time interval of 480 s and a step size of 0.002 cm. Multiple scans of sedimentation velocity data were collected and analyzed using the software SEDFIT 9.4c.

### Computer methods

Computer methods were used to predict the *gsmt* and *sdmt* amino acid sequences along with BLAST from the NCBI web site (http://www.ncbi.nlm.nih.gov/BLAST/) to compare protein sequences. Multiple sequence alignments were constructed using the SDSC Biology Workbench website (http://workbench.sdsc.edu) with the ClustalW program [27]. Phylogenetic and molecular evolutionary analyses were conducted using MEGA version 3.1 [28].

## Results and Discussion

### Betaine synthesizing *gsmt* and *sdmt* genes and the associated *M. portucalensis* gene cluster

**Figure 1:**
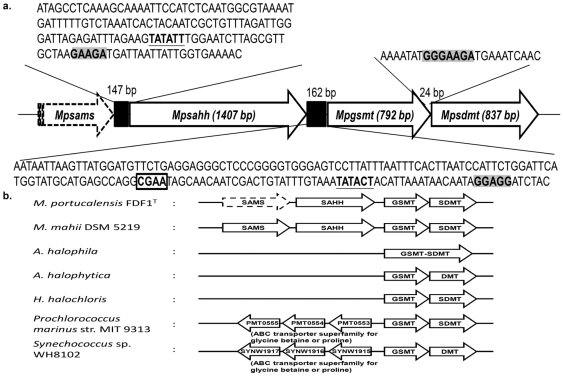
Gene analysis of *gsmt* and *sdmt*. a. The predicted TATA box (underline), ribosome binding site (shadow) and heat-shocked regulatory element (rectangle) located at the upstream of the *Mpsahh*, *Mpgsmt* and *Mpsdmt*. b. Comparison of the gene arrangement of known betaine *de novo* synthesizing genes. Upstream of the *Mpgsmt-sdmt* gene cluster was the AdoHcy hydrolase (SAHH) and AdoMet synthetase (SAMS) genes related to the osmolyte betaine biosynthesis pathway. In contrast to whole genome sequencing database of *P. marinus* str. MIT9313 and *S.* sp WH8102, upstream of the *gsmt* gene was the betaine/proline ABC transporter. In our knowledge, there is no information of what gene is upstream the *gsmt* in *A. halophila*, *A. halophytica*, and *H. halochloris*.

A 3.9 Kb fragment of *M. portucalensis* was obtained from Southern hybridization as described in the Materials and Methods that contain the partial S-adenosylmethionine synthetase (*sams*) gene fragment, the full-length S-adenosyl homocysteine hydrolyase (*sahh*) genes, glycine sarcosine methyltransferase (*gsmt*), and sarcosine dimethylglycine methyltransferase (*sdmt*) genes. The complete *Mpsahh* gene is 1407 bp with an estimated %G+C content of 45.00% and encoded by 468 amino acids. The complete *Mpgsmt* (HQ634197) gene is 792 bp with an estimated %G+C content of 43.18% and encoded by 263 amino acids. The complete *Mpsdmt* (HQ634198) gene is 837 bp with an estimated %G+C content of 42.17% and encoded by 278 amino acids. A putative TATA box (TATACT) and ribosome binding site (GGAGG) upstream of *Mpgsmt* were detected (Fig. 1a), and a putative ribosome binding site (GGGAAGA) was also found upstream of *Mpsdmt* (Fig. 1a).

**Figure 2:**
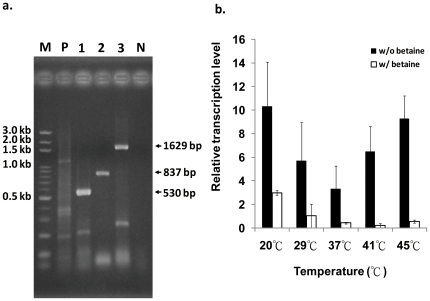
Analysis the transcription unit and transcription level of *Mpgsmt-sdmt* under temperature stresses. a. RT-PCR analysis of *Mpgsmt* and *Mpsdmt* expression in *M. portucalensis*. M: Gen-100 DNA ladder; 1. RT-PCR product of partial *Mpgsmt* (530 bp); 2. RT-PCR product of *Mpsdmt* (837 bp); 3. RT-PCR product of *Mpgsmt-sdmt* (1629 bp); P: 16s rRNA (1200 bp) as positive control; N: RT-PCR reaction without RTase addition as negative control. b. The temperature effect on the transcription of *Mpgsmt-sdmt*. The white and black bars represent the samples with or without the addition of 0.5 mM glycine betaine, respectively. Data were obtained from the average of three independent tests.

Gene arrangement suggested that these two betaine synthesis-associated genes may belong to the same gene cluster (Fig. 1a). To further determine the transcripts of the *Mpgsmt-sdmt* gene cluster, the cDNAs of partial *Mpgsmt*, *Mpsdmt*, and *Mpgsmt-sdmt* fragments were amplified by RT-PCR using *M. portucalensis* total RNA as a template with the specific primers Mpgsmt/NdeI-f, Mpgsmt-rw, Mpsdmt/NdeI-f, and Mpsdmt/XhoI-r. As shown in Fig. 2a, the expected partial *Mpgsmt* (530 bp), *Mpsdmt* (837 bp), and *Mpgsmt-sdmt* (1653 bp) products were amplified. The co-transcription of *Mpgsmt-sdmt* was confirmed by Northern hybridization with *M. portucalensis* RNA and probes *Mpgsmt*, *Mpsdmt*, and *Mpgsmt-sdmt* (Fig. S1). These results verified that *Mpgsmt* and *Mpsdmt* were cotranscribed.

The CGAA element, which is located upstream of the putative *Mpgsmt* TATA box, was detected (Fig. 1a). This element is a necessary DNA regulator for heat shock proteins in several archaea [29]. The occurrence of the CGAA element upstream of *Mpgsmt* suggested that the expression of the betaine synthesizing gene cluster *Mpgsmt-sdmt* would be affected by heat shock. Using a mid-log culture of *M. portucalensis* FDF1^T^, which was grown at 37°C, the relative transcription level of *Mpgsmt-sdmt* was analyzed under temperature shock conditions, that is, either high or low, for 1 h. The transcription level of *Mpgsmt-sdmt* was up-regulated by both heat (41, 45°C) and cold (20, 29°C) stresses (Fig. 2b). However, upon external addition of betaine, the transcription level of *Mpgsmt-sdmt* was significantly decreased (Fig. 2b). This result suggests that betaine functions not only as an osmolyte in halophilic methanogens but also as a thermo- and cryo-protectant.

Genes located upstream of the *M. portucalensis* FDF1^T^ *Mpgsmt-sdmt* are encoded by S-adenosyl homocysteine hydrolyase (*Mpsahh*) and S-adenosyl-methionine synthetase (*Mpsams*) (Fig. 1a). S-adenosyl-methionine synthetases can synthesize AdoMet, which is the methyl donor for GSMT and SDMT. S-adenosyl homocysteine (AdoHcy) is a strong inhibitor of GNMT, GSMT and SDMT [20]. S-adenosyl homocysteines hydrolase can hydrolyze AdoHcy to adenosine and homocysteine and reduce the inhibition of that molecule. Compared with the gene arrangement of known betaine *de novo* synthesizing genes from the Bacteria (Fig. 1b), genes upstream of the *gsmt* from *Synechococcus* sp. WH 8102 and *Prochlorococcus marinus* str. MIT 9313 are all related to the betaine/proline ABC transporter, which transports extracellular betaine or proline. Compare with the whole genome sequenced *M. mahii* DSM 5219 [30], it had the same gene arrangement with *M. portucalensis* which described above. To our knowledge, this is the first report showing that the AdoHcy hydrolase and AdoMet synthetase genes are located upstream of the *gsmt-sdmt* gene cluster in *Methanohalophilus* genus. This gene arrangement suggested that the expression of *sams* and *sahh* may associate with the regulatory mechanism of the betaine biosynthesis.

### *M. portucalensis* GSMT and SDMT

The estimated molecular masses of the *M. portucalensis* GSMT and SDMT are 29,040 and 30,690 Da, respectively, which are both relatively smaller than those from halotolerant and halophilic bacteria, 32,940 and 31,580 Da, respectively [15]–[20]. The estimated p*I* value of MpGSMT is 4.57, which is lower than for other known GSMTs from halophilic bacteria (p*I* from 4.67–5.47). However, the p*I* values of all known SDMTs are in a similar range (4.67–5.82).

**Figure 3:**
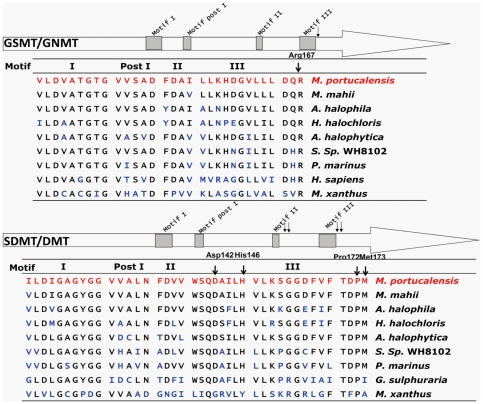
Comparison of AdoMet binding motifs and substrate binding sites from gsmt or sdmt/dmt. Sequences of *Mpgsmt* and *Mpsdmt* were aligned with *gsmt/gnmt* and *sdmt*/*dmt* from other organisms including *A. halophila* (AAF87204); *A. halophytica* (ABO94497, ABO94498); *H. halochloris* (AAF87202, AAF87203); *G. sulphuraria* (Gs07580.1); *P. marinus* str. MIT9313 (CAE20427, CAE20426); *S.* sp. WH8103 (CAE08429, CAE08428). I, postI, II, III indicate the AdoMet binding motif I, postI, II, III, respectively. The arrows showed the substrate binding sites.

The putative substrate- (glycine, sarcosine and dimethylglycine) binding sites and conserved AdoMet-binding motif I, post I, II and III of MpGSMT and MpSDMT are listed in Fig. 3 along with the other known betaine synthesis-related N-methyltransferases. Although the total amino acid identity between GSMT and mammalian GNMT is low, AdoMet-binding motif I, post I and II are highly conserved. Site-directed mutagenesis studies have shown that the conserved Arg169Glu of the *A. halophytica* GSMT and the conserved Pro171Gln and Met172Arg of *A. halophytica* DMT are involved in methyl acceptor binding [20]. Crystal structure studies of GsSDMT showed that the conserved Asp158 and His162 of GsSDMT (Fig. 3) may directly interact with the substrates sarcosine and dimethylglycine [31]. The *Mp*GSMT Arg167 is analogous to the conserved *A. halophytica* GSMT Arg169 that may be involved in binding glycine or sarcosine. Pro172, Met173, Asp142 and His146 of the *Mp*SDMT, which correspond to *A. halophytica* DMT Pro171 and Met172 and GsSDMT Asp158 and His162, may function as sarcosine- or dimethylglycine-binding sites. These highly conserved motifs and substrate-binding residues imply that MpGSMT and MpSDMT are glycine, sarcosine and dimethylglycine methyltransferases.

### Phylogenetic analyses of GSMT and SDMT

The amino acid sequences of the *M. portucalensis* GSMT and SDMT are related to the *M. mahii* GSMT and SDMT (95% and 91% amino acid identities, respectively). The identities of the MpGSMT compared with other GSMTs from halophilic bacteria are within 61–66%. When the MpSDMT was aligned with homologs from other halophilic bacteria, the identities ranged from 48% to 58%. In contrast, the homology between the MpGSMT and the *H. sapiens* GNMT was low (32% identity).

**Figure 4:**
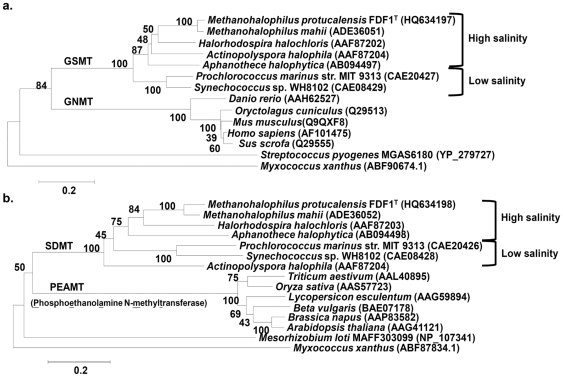
Phylogenetic relationship of GSMT (a) and SDMT (b) with related N-methyltransferase. Bootstrap values are shown at nodes (percentage of 100 replicates). Bar, 0.1 aa substitutions per 100 aa.

A phylogenetic tree was constructed for the MpGSMT using other halophilic bacterial GSMTs or mammalian GNMTs (Fig. 4a). GSMTs from bacteria and archaea and GNMTs from mammals clustered separately, which indicated that GSMTs and GNMTs may have differentially evolved. The phylogenetic relationship of MpSDMT to related homologs was also investigated as shown in Fig. 4b. On the basis of this phylogenetic analysis, both GSMTs and SDMTs from bacterial and archaeal sources were separated into two clusters that were related to the optimal salinities of these organisms. This observation indicates that the evolution of GSMT and SDMT might be diversified based on the salinity of the environments occupied by these organisms.

### *In vivo* betaine accumulation in *E. coli* BL21(DE3)RIL and MKH13

**Figure 5:**
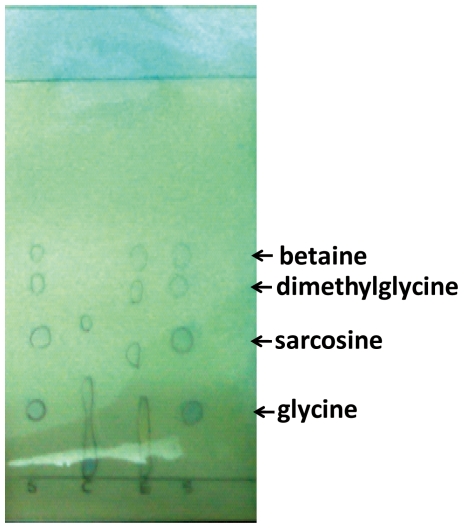
The intracellular solutes from *E. coli* BL 21(DE3)RIL-pET28a-*Mpgsmt-sdmt* separated by TLC silica plate. S, 2 mmole of glycine, sarcosine, dimethylglycine and betaine as standard; C, solutes of *E. coli* BL21(DE3) RIL–pET28a as negative control; E, solutes of *E. coli* BL21(DE3)RIL-pET28a-*Mpgsmt-sdmt*. The TLC plate is stained by 0.1% bromocresol green.

The *in vivo* activities of recombinant MpGSMT and MpSDMT were evaluated. Intracellular solutes were extracted from *E. coli* BL21(DE3)RIL-pET28a-*Mpgsmt-sdmt* and separated on a thin-layer chromatography (TLC) silica plate using a phenol-water (4∶1) solvent system. As shown in Fig. 5, ethanol extracts from *E. coli* BL21(DE3) RIL-pET28a-*Mpgsmt-sdmt* showed accumulations of sarcosine, dimethylglycine, and betaine, and the *E. coli* control did not. This result indicated that the recombinant MpGSMT and MpSDMT both expressed their functional activities in *E. coli* BL21(DE3)RIL.

**Figure 6:**
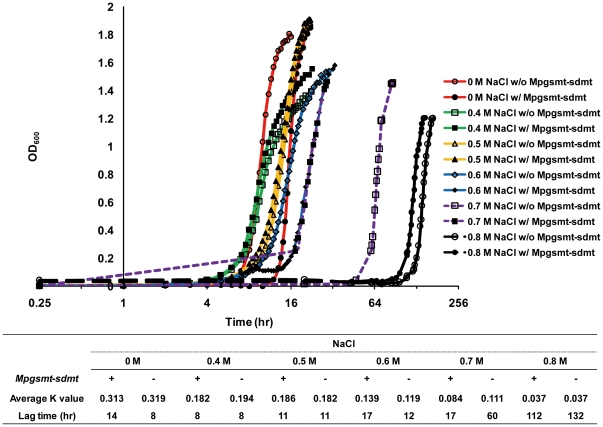
The growth of *E. coli* MKH13 with or without the co-expression of MpGSMT and MpSDMT. Each sample was sub-transferred at least three times to adapt the various salinity environments before the growth measurement. Logarithm phase cultures (OD_600_ obtained 0.86) were sub-transferred 1 ml to 100 ml fresh M9 minimal medium in the same standard 500 ml flasks and incubated at 37°C under 250 rpm shaking. The absorbance of all cultures was measured by spectrophotometer with 600 nm wavelength. Symbolism as follows: red line with circle mark, 0 M NaCl; green line with square mark, 0.4 M NaCl; yellow line with triangle mark, 0.5 M NaCl; blue line with diamond mark, 0.6 M NaCl; purple spot line with square mark, 0.7 M NaCl; black dash line with circle mark, 0.8 M NaCl. Full symbols and hollow symbols indicated with or without *Mpgsmt-sdmt* gene cluster respectively. The accompanying table shows the lag time and the average K value as growth rate of different cultures.

To further verify that the functional expression of the *Mpgsmt* and *Mpsdmt* genes could enhance survival of the host *E. coli* in a high salt environment, growth tests that utilized a betaine transport-deficient mutant *E. coli* MKH13 with both *Mpgsmt* and *Mpsdmt* genes under different salinities were tested. In high salt media (0.7 and 0.8 M NaCl), the lag time of *E. coli* MKH13 that co-expressed MpGSMT and MpSDMT was reduced (Fig. 6). This result indicated that co-expression of MpGSMT and MpSDMT could help the host *E. coli* MKH13 to tolerate the high salt stress.

### Kinetic properties of recombinant MpGSMT and MpSDMT

**Figure 7:**
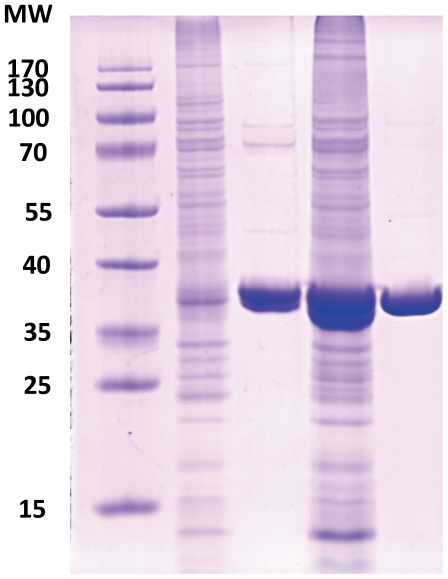
SDS-PAGE of heterologous expressed and purified MpGSMT and MpSDMT. M: PageRuler™ pre-stained protein ladder; G_CE_: crude extract of MpGSMT; G: purified MpGSMT; S_CE_: crude extract of MpSDMT; S: purified MpSDMT. For each lane, total 10 µg quantiated protein was loaded and the SDS-PAGE was stained by coomassie blue.

MpGSMT and MpSDMT were separately expressed in *E. coli* BL21(DE3)RIL under the control of the T7 promoter. Conditions for vector construction, and overproduction and purification of heterologously expressed proteins are described in the Materials and Methods. The molecular masses of purified MpGSMT (35 kD) and MpSDMT (38 kD) were analyzed by electrophoresis (Fig. 7). MpGSMT and MpSDMT purities were 90% and 95%, respectively.

The methyl transfer reactions of recombinant MpGSMT and MpSDMT were measured by the acid-washed charcoal method. The K_m_ and V_max_ values are listed in Table 1. The catalytic efficiencies of recombinant MpGSMT (8.19×10^−8^ for GMT, 1.09×10^−5^ for SMT) were extraordinarily low, which may be due to the high K_m_ values, namely, 2.32 M for GMT and 3.02 M for SMT, while known halophilic bacteria have K_m_ values in the mM range (0.5 to 18.0 mM) for GSMT (Table 1; [16], [18]–[20]). However, the catalytic efficiencies of recombinant MpSDMT were 0.67 for SMT and 1.12 for DMT with K_m_ values of 2.29 mM for SMT and 3.76 mM for DMT, which were in the range of other heterologous SDMT/DMT from halotolerant and halophilic bacteria (Table 1).

**Table 1:** Kinetic analysis of betaine de novo synthesizing enzymes.

Resource	Enzyme		Apparent K_m_ (mM)				V_max_	K_cat_/K_m_	Reference
			Gly.	Sar.	Dim.	AdoMet	(nmol µg^−1^ min^−1^)		
*Aphanothece halophytica*	GSMT	GMT	1.50			0.70	0.500	0.833	Waditee et al., 2003
		SMT		0.80		0.60	0.290	0.906	
	DMT	DMT			0.50	0.18	0.470	2.350	
*Galdieria sulphuraria*	SDMT	SMT		2.00		ND	ND	32.150	McCoy et al., 2009
		DMT			2.80	0.15	ND	30.571	
*Halorhodospira halochloris*	GSMT	GMT	18.00			0.42	1.100	0.153	Nyyssölä et al., 2001
		SMT		2.30		0.28	0.140	0.152	
	SDMT	SMT		6.10		0.21	1.200	0.492	
		DMT			4.90	0.16	6.800	3.469	
*Methanohalophilus portucalensis*	GSDMT*	GMT	0.47			0.47	0.002	0.010	Lai et al., 2006
		SMT		3.14		ND	0.006	0.005	
		DMT			2.45	ND	0.004	0.004	
	SDMT*	SMT		2.29		0.21	0.810	0.884	Chen et al., 2009
		DMT			3.76	0.59	4.880	3.244	
	GSMT	GMT	2320.00			8.26	0.019	8.19×10^−8^	This study
		SMT		3021.00		1.78	0.033	1.09×10^−5^	
	SDMT	SMT		0.66		0.50	0.440	0.670	
		DMT			0.66	0.79	0.740	1.120	
*Synechococcus* sp. WH8102	GSMT	GMT	5.22			0.30	1.480	0.709	Lu et al., 2006
		SMT		2.94		0.44	0.780	0.663	
	DMT	DMT			2.11	0.41	1.180	1.398	

*proteins purified from original cell.

The K_m_ values for the methyl donor AdoMet for both recombinant GSMT (8.26 mM for GMT and 1.78 mM for SMT) and SDMT from *M. portucalensis* (0.50 mM for SMT and 0.79 mM for DMT) were in a similar range compared with other GSMTs and SDMTs/DMTs from halotolerant and halophilic bacteria, which are in the mM range.

It is common for the recombinantly expressed enzyme to have a lower catalytic efficiency compared with the enzyme isolated from the original cells. For recombinant MpGSMT, the nearly 10,000-fold decrease in catalytic efficiency is significant and is mainly due to the low affinity for binding methyl acceptors. One possible contribution may have been due to the occurrence of an abnormal structure around the substrate glycine or the sarcosine-binding domains, which would increase the K_m_ of the heterologously expressed MpGSMT.

### The effect of inorganic osmolytes (potassium and sodium), organic osmolyte betaine and methyltransferase inhibitor AdoHcy on the kinetic properties of recombinant MpGSMT and MpSDMT

Intracellular potassium concentrations of 0.6–1.0 M in *M. portucalensis* strain FDF1^T^ have been reported [12], and a concentration of up to 3.0 M K^+^ in a related extremely halophilic methanogen grown in 4.1 M NaCl has been documented [13]. This information suggests that halophilic methanogens use dual strategies, namely, compatible solute (osmolyte) accumulation and salting-in [3], to adapt to high salt environments. *In vitro* betaine synthesis by *M. portucalensis* assayed in cell extracts showed that sarcosine accumulation was regulated by monovalent cations (lithium, sodium and potassium) [5].

**Figure 8:**
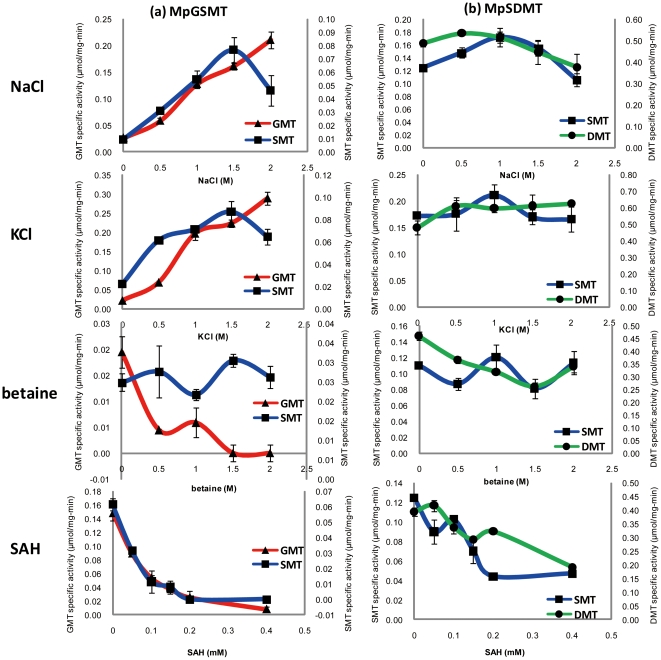
The effects of sodium, potassium, betaine, and AdoHcy on MpGSMT and MpSDMT. A. Methyltransferase activities of glycine methyltransferase (GMT,▴) and sarcosine methyltransferase (SMT, ▪) on MpGSMT. B. Methyltransferase activities of sarcosine methyltransferase (SMT, ▪) and dimethylglycine methyltransferase (DMT,•) on MpSDMT. All the specific activities of methyltransferases were averaged from independent triplicate acid-washed charcoal assays as described in Materials and Methods.

The effect of inorganic osmolyte potassium and sodium concentrations on the methyltransferase activity of MpGSMT was dramatic. The GMT activity of MpGSMT was enhanced 9.21- and 13.50-fold with 1.0 M and 2.0 M KCl, respectively, and enhanced 4.91- and 8.10-fold with 1.0 and 2.0 M NaCl, respectively. The MpGSMT SMT was enhanced 3.19- and 2.89-fold with 1.0 and 2.0 M KCl, respectively, and 5.80- and 4.91-fold with 1.0 and 2.0 M NaCl, respectively (Fig. 8; Table 2). These data are consistent with previous *in vitro* assays of betaine synthesis by *M. portucalensis* FDF1^T^ crude extracts [5]. Additionally, higher levels of monovalent cations could enhance the substrate-binding affinity of MpGSMT. The K_m_ values for the GMT of MpGSMT at potassium levels of 0 and 2.0 M were 2.74 M and 55.0 mM, respectively, and for SMT of MpGSMT at 0 and 2.0 M potassium ions conditions with 3.71 M and 90.0 mM K_m_ values, respectively (unpublished data). The above experimental results suggest that the levels of monovalent cations play an important role in modulating the substrate-binding affinity of MpGSMT. However, the specific activities of GMT and SMT for the GSMT from the halophilic bacteria *A. halophytica* were both inhibited to 50.0% with 1.0 M KCl and inhibited to 83.0% and 30.0%, respectively, with 1.0 M NaCl [20]. The specific activities for the GSMTs from halophilic archaea increased with an increasing level of K^+^ or Na^+^. This finding indicated that the first enzymatic step in the synthesis of the betaine osmolyte by GSMTs from halophilic archaea could be up-regulated by potassium or sodium ions. GSMTs may play a major role in coupling the salt-in and compatible solute (osmolyte) osmoadaptative strategies in halophilic methanogens to adapt to high salt environments.

**Table 2:** Effects of NaCl, KCl, betaine and AdoHcy on recombinant MpGSMT and MpSDMT activities.

Enzyme	Methyl group acceptor (mM)	Relative activity (%)							I_50_ AdoHcy (mM)
		No addition	NaCl		KCl		Betaine		
			1 M	2 M	1 M	2 M	1 M	2 M	
MpGSMT	Glycine (1000)	100	491	810	921	1353	30	0	0.07
	Sarcosine (1000)	100	580	491	319	289	88	105	0.05
MpSDMT	Sarcosine (1000)	100	138	85	122	96	109	103	0.15
	Dimethylglycine (1000)	100	106	77	124	130	69	74	0.40

In contrast to MpGSMT, the methyltransferase activity of recombinant MpSDMT was slightly inhibited by potassium and sodium ions. The specific activities of the recombinant MpSDMT were not inhibited by potassium ions, but slightly inhibited to 85.0% and 77.0% for SMT and DMT, respectively, with 2.0 M NaCl (Fig. 8; Table 2). The *A. halophytica* DMT activity was inhibited to 50.0% with 1.0 M KCl and inhibited to 83.0% with 1.0 M NaCl [20]. The effect of the sodium and potassium levels on SDMT or DMT activity in both halophilic archaea and bacteria was similar.

Feedback inhibition on the methyl transfer reactions of recombinant MpGSMT and MpSDMT by the betaine end product was investigated. The methyltransferase activities were analyzed after the addition of 0 to 2.0 M betaine. For recombinant MpGSMT, the GMT-specific activities were significantly inhibited and only 30.0% and 0% of the residual activities remained after the addition of 1.0 and 2.0 M betaine, respectively. However, the SMT activities were not affected by betaine (Fig. 8; Table 2). The MpSDMT specific activity was also not affected by betaine (Fig. 8; Table 2). In contrast, the specific activities of both the GSMT and DMT from *A. halophytica* were not affected by 2.0 M betaine [20]. Inhibition of the first enzymatic step in *de novo* betaine synthesis, the GMT activity of MpGSMT, by the betaine end product prevented buildup of intermediates and terminated the methyl transfer reaction to economize energy.

AdoMet-dependent methyltransferases are strongly inhibited by S-adenosyl- L-homocysteine (AdoHcy). AdoHcy inhibition on bacterial GSMT and SDMT/DMT activities show K_i_ values in a range of 0.3 to 2.3 mM [19], [20], which makes AdoHcy a potent regulator. The inhibition of recombinant MpGSMT and MpSDMT was tested with 0.05 to 0.40 mM AdoHcy. As shown in Fig. 8, both MpGSMT and MpSDMT activities were inhibited by increasing AdoHcy concentrations. The level of AdoHcy for 50.0% inhibition of MpGSMT activity was 0.05 mM and 0.07 mM for GMT and SMT, respectively. About 50.0% MpSDMT inhibition was achieved by AdoHcy concentrations of 0.15 and 0.40 mM for SMT and DMT activities, respectively. The methyltransferase activities of MpGSMT were more sensitive to AdoHcy compared with MpSDMT.

The halophilic methanogen *M. portucalensis* FDF1^T^ can accumulate 1.1 M of potassium ions as primary response inorganic osmolytes and can also synthesize and accumulate 0.65 M betaine as an organic osmolyte when grown with 2.7 M of external NaCl [12]. *Methanohalophilus* strain Z7302 can also accumulate 3.1 M of potassium ions and *de novo* synthesize 3.9 M betaine when grown in a 4.1 M NaCl environment [13]. The methyl transfer activity of MpGSMT showed that it could be induced by the addition of potassium or sodium ions (Fig. 8; Table 2). The higher level of potassium or sodium ions enhanced the methyl acceptor glycine or sarcosine-binding affinity of MpGSMT. However, the secondary structure of MpGSMT, which was estimated by circular dichroism, showed no affect because of the increase in potassium or sodium ions from 0 to 2.0 M (Lai & Lai, unpublished data). Nevertheless, a fluctuated peak at 206 nm was detected in the three independent experiments in the MpGSMT CD spectra under various concentrations of potassium ions (Fig. S2). This fluctuated difference may have been caused by a change in the MpGSMT β-turn structure [32] under the influence of increasing concentrations of potassium ions. The MpGSMT β-turn structure predicted by PSIPRED (http://bioinf.cs.ucl.ac.uk/psipred/) was located at the conserved glycine- and sarcosine-binding residue Arg167 of MpGSMT (Fig. 3), but this structure was not detected in MpSDMT. Quaternary structure analyses of MpGSMT under different concentrations of KCl were carried out by analytical ultracentrifugation. The monomer MpGSMT switched to dimeric form increased from 7.6% to 70% with KCl concentration increased from 0 to 2.0 M (Fig. S3). This conformational change under different salt concentration may affect the substrate-binding affinity of MpGSMT and the specific methyltransfer activities were significantly activated by a high concentration of potassium or sodium ions. Compared with GSMTs from halophilic bacteria, this characteristic of the halophilic archaeal MpGSMT is unique and increased methyltransferase activities with increasing salt concentrations, while other GSMT activities were inhibited by increasing salt concentrations [18], [20]. The results reported in this study suggest that internal potassium levels and MpGSMT play a major role in betaine *de novo* synthesis and accumulation under hyperosmotic stress.

In *M. portucalensis* FDF1^T^, betaine accumulation to overcome hyperosmotic stress can occur by primary ABC betaine transporter or biosynthesis by a three-step methylation process. The supply of the methyl donor AdoMet through the activity of the AdoMet synthetase and the degradation of the inhibitor AdoHcy by the AdoHcy hydrolase should be important regulatory mechanisms for betaine accumulation *de novo*. To continue to synthesize and accumulate betaine in cells, it is important to efficiently remove the AdoHcy inhibitor. In the halophilic methanogen *M. portucalensis*, the AdoHcy hydrolase gene is located upstream of the *Mpgsmt-sdmt* gene cluster. This location may suggest that the AdoHcy hydrolase also plays a role in regulating betaine synthesis and accumulation in cells. *M. portucalensis* is the first organism where the betaine synthesizing genes *gsmt* and *sdmt* are clustered with the AdoMet synthetase gene and the hydrolase that decomposes the AdoHcy inhibitor. The placement of these genes suggests that the transcription levels or protein activities of the AdoMet synthetase and AdoHcy hydrolase could be induced under hyperosmotic stress to supply and ensure betaine synthesis. This novel gene arrangement and regulation for betaine synthesis by halophilic archaea is of interest for further study.

In summary, we have cloned and characterized the betaine synthesizing gene cluster *gsmt-sdmt* and *sahh* from *M. portucalensis*. As betaine *de novo* synthesizing enzymes from halophilic bacteria, the recombinant MpGSMT and MpSDMT could methyl transfer glycine and sarcosine or sarcosine and dimethylglycine, respectively, to accumulate betaine. The dramatic activation effects of sodium and potassium ions and inhibitory effect of betaine and AdoHcy on MpGSMT, but not MpSDMT, indicated that MpGSMT was the key enzyme for regulating the betaine synthesis pathway in *M. portucalensis* FDF1^T^. And the potassium level (internal potassium concentration as in vivo) may modulate the glycine and sarcosine methyl transfer activities of MpGSMT through conformational change from monomeric to dimeric form. The accumulation of betaine and the intermediates sarcosine and dimethylglycine in *E. coli* BL21(DE3)RIL-pET28a-*Mpgsmt-sdmt* indicated that the heterologously expressed halophilic archaeal GSMT and SDMT indeed had *in vivo* activities. Co-expression of MpGSMT and MpSDMT could help the host *E. coli* MKH13 to resist high salt stresses (0.7 and 0.8 M NaCl). This is the first report that the *de novo* betaine synthesis process could be activated by a rapid accumulation of potassium ions, which is a primary response to hyperosmotic stress, and deactivated by a high concentration of the betaine end product, which shuts off transcription of *Mpgsmt-sdmt* and inhibits the methyl transfer activity of MpGSMT. These results imply that the betaine *de novo* synthesis pathway in *M. portucalensis* FDF1^T^ is a process to economize energy to survive under hyperosmotic stress.

## Supporting Information

Figure S1 **Northern hybridization with probes of** ***Mpgsmt*** **,** ***Mpsdmt*** **or** ***Mpgsmt-sdmt*** **separately revealed the transcripts of** ***Mpgsmt*** **and** ***Mpsdmt*** **in** ***M. portucalensis*** **.** Total RNA from three independent cultures of *M. portucalensis* FDF1^T^ was loaded onto different lane (lane 1∼3) with triplicate. After formaldehyde gels electrophoresis, the RNA was transferred to Nylon membrane and cut as three separate gels for hybridization with probes *Mpgsmt*, *Mpsdmt* or *Mpgsmt-sdmt*, respectively. The arrow indicated the major signal of *Mpgsmt-sdmt* which hybridized with probe indicated at top panel. Other unexpected signals on membrane may due to the non-specific hybridization by the long full gene probes used in this study.
(TIF)

Figure S2 **Circular-dichroism spectra of MpGSMT with addition of diverse level of KCl.** The arrow shows the fluctuated peak at 206 nm wavelength.
(TIF)

Figure S3 **Quaternary structure analyses of MpGSMT under different concentrations of KCl by analytical ultracentrifugation.** The enzyme concentration used in the experiments was 0.70 mg/ml in 0.1 M TES buffer (pH 7.3) at 20°C. Multiple scans of data were collected and analyzed by software SEDFIT 9.4c.
(TIF)
