# Supplementary figures and images for: Characterization and Regulation of the Osmolyte Betaine Synthesizing Enzymes GSMT and SDMT from Halophilic Methanogen Methanohalophilus portucalensis
Source: PLoS One. 2011 Sep 20;6(9):e25090. doi: 10.1371/journal.pone.0025090 (PMC3176816; doi:10.1371/journal.pone.0025090)

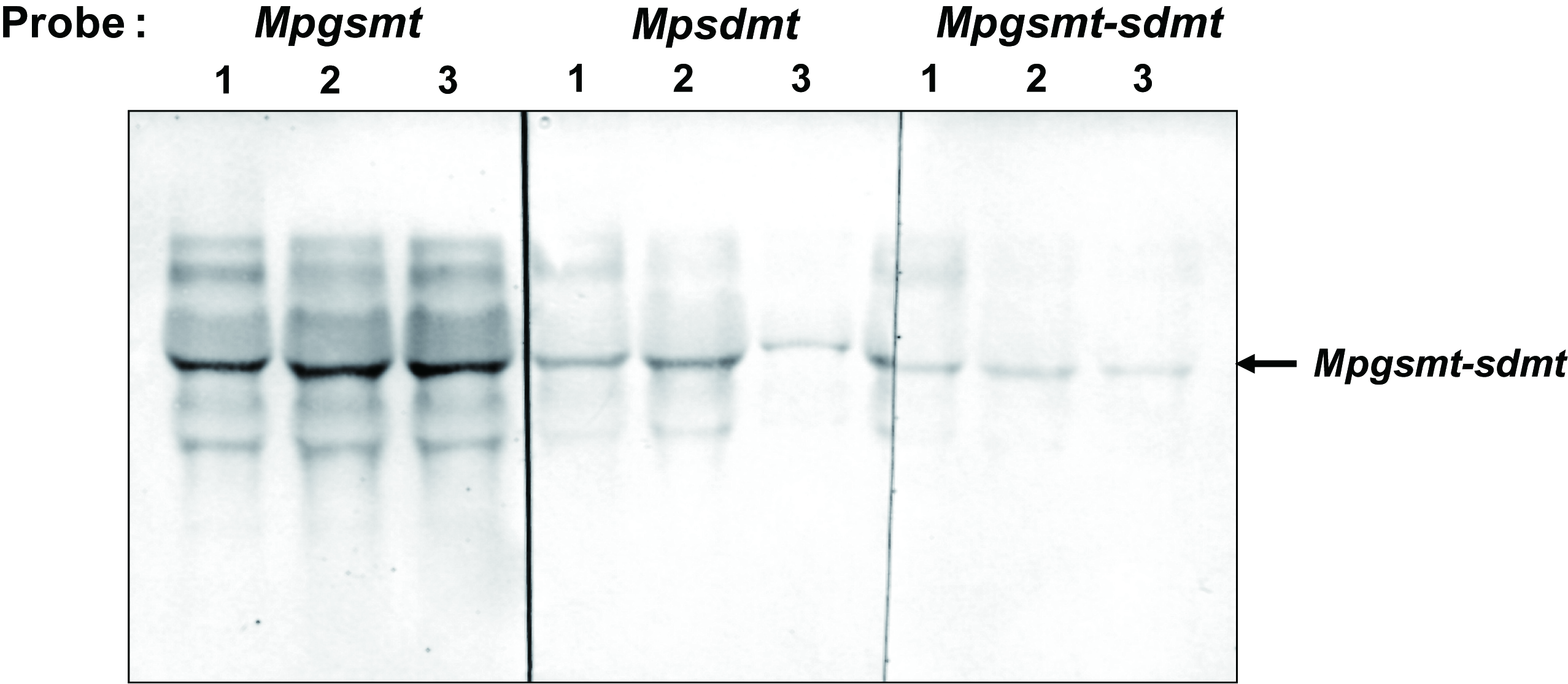

Supplement: Figure S1 — Northern hybridization with probes of Mpgsmt , Mpsdmt or Mpgsmt-sdmt separately revealed the transcripts of Mpgsmt and Mpsdmt in M. portucalensis . Total RNA from three independent cultures of M. portucalensis FDF1T was loaded onto different lane (lane 1∼3) with triplicate. After formaldehyde gels electrophoresis, the RNA was transferred to Nylon membrane and cut as three separate gels for hybridization with probes Mpgsmt, Mpsdmt or Mpgsmt-sdmt, respectively. The arrow indicated the major signal of Mpgsmt-sdmt which hybridized with probe indicated at top panel. Other unexpected signals on membrane may due to the non-specific hybridization by the long full gene probes used in this study. (TIF) [file pone.0025090.s001.tif]

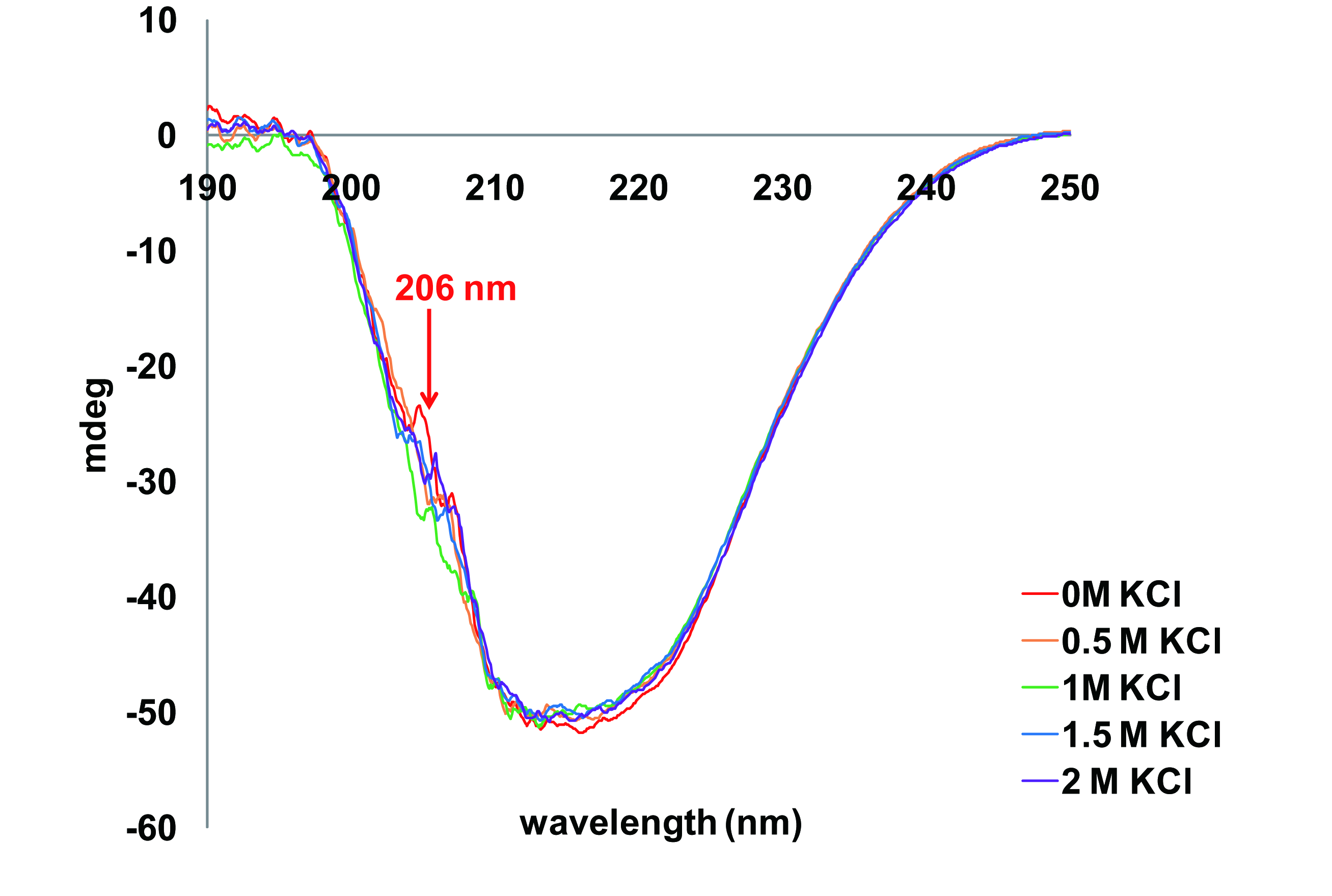

Supplement: Figure S2 — Circular-dichroism spectra of MpGSMT with addition of diverse level of KCl. The arrow shows the fluctuated peak at 206 nm wavelength. (TIF) [file pone.0025090.s002.tif]

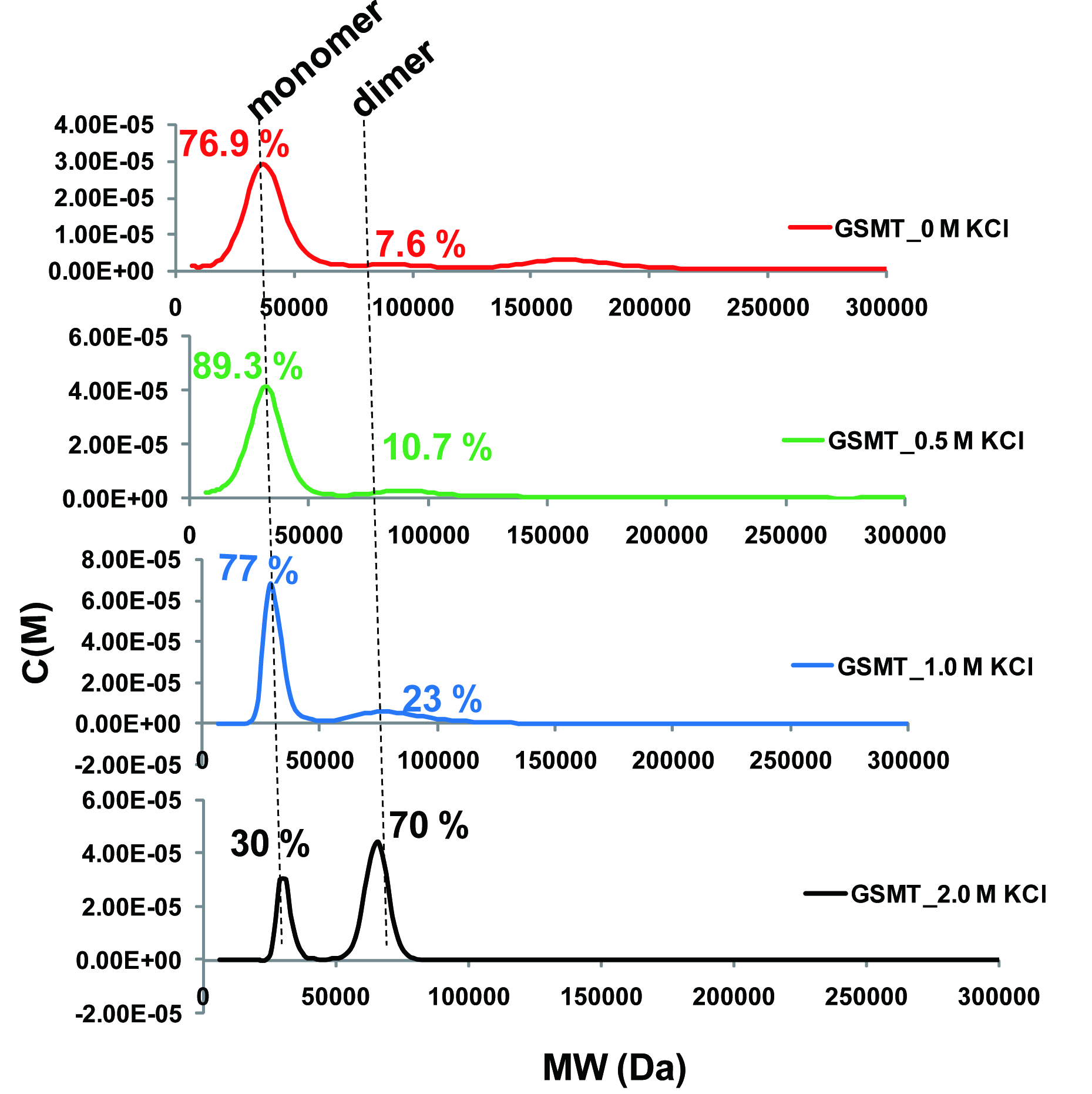

Supplement: Figure S3 — Quaternary structure analyses of MpGSMT under different concentrations of KCl by analytical ultracentrifugation. The enzyme concentration used in the experiments was 0.70 mg/ml in 0.1 M TES buffer (pH 7.3) at 20°C. Multiple scans of data were collected and analyzed by software SEDFIT 9.4c. (TIF) [file pone.0025090.s003.tif]
